# Marine Alkylpurines: A Promising Group of Bioactive Marine Natural Products †

**DOI:** 10.3390/md16010006

**Published:** 2018-01-01

**Authors:** Pablo A. García, Elena Valles, David Díez, María-Ángeles Castro

**Affiliations:** 1 Department of Pharmaceutical Sciences, Medicinal Chemistry Section, Pharmacy Faculty, CIETUS, IBSAL, University of Salamanca, E-37007 Salamanca, Spain; pabloagg@usal.es (P.A.G.); elenitavm@usal.es (E.V.); 2 Department of Organic Chemistry, Faculty of Chemical Sciences, University of Salamanca, E-37008 Salamanca, Spain; ddm@usal.es

**Keywords:** marine natural products, purines, methylpurines, alkylpurines, terpenylpurines, cytotoxicity, antimicrobial activity

## Abstract

Marine secondary metabolites with a purine motif in their structure are presented in this review. The alkylpurines are grouped according to the size of the alkyl substituents and their location on the purine ring. Aspects related to the marine source, chemical structure and biological properties are considered together with synthetic approaches towards the natural products and bioactive analogues. This review contributes to studies of structure–activity relationships for these metabolites and highlights the potential of the sea as a source of new lead compounds in diverse therapeutic fields.

## 1. Introduction

Natural products play a highly significant role in drug discovery and development processes. It is well known that a high proportion of commercial drugs came from natural compounds isolated from plants and terrestrial organisms [1]. In this sense, the sea is a relatively unexplored source of natural products, which offers a significant potential for the discovery of molecules with relevance in different therapeutic areas. These natural products are isolated from many different organisms and show a wide structural variety [2,3].

Among all the natural products that can be isolated from the marine environment, this article focussed solely on marine secondary metabolites containing a purine moiety. Purines are the most widely naturally occurring heterocycles and exist as free bases, nucleosides, nucleotides, polynucleotides, or structural fragments of vitamins [4], but we will focus our attention on those formed by a purine unit substituted with different alkyl chains at any nitrogen atom of the purine. These compounds have been organized according to the size and nature of the alkyl chain, ranging from simple methyl groups to larger side chains.

In this article, aspects related to the marine source, chemical structure and biological properties of marine alkylpurines are considered together with some synthetic approaches towards the natural products and/or their bioactive analogues.

## 2. Purines Alkylated Only with Methyl Groups

The simplest alkyl substituent that can be found on the purine ring is the methyl group. Usually, the purines isolated from marine organisms contain two or three methyl groups attached to any nitrogen atoms in the heterocycle core, including the amino groups that can be present at C2 or C6, being less frequent the monomethylated derivatives. Most are derived from adenine, guanine, isoguanine or 8-oxopurine, with a few xanthine derivatives.

### 2.1. Adenine and Guanine Derivatives

**Figure 1:**
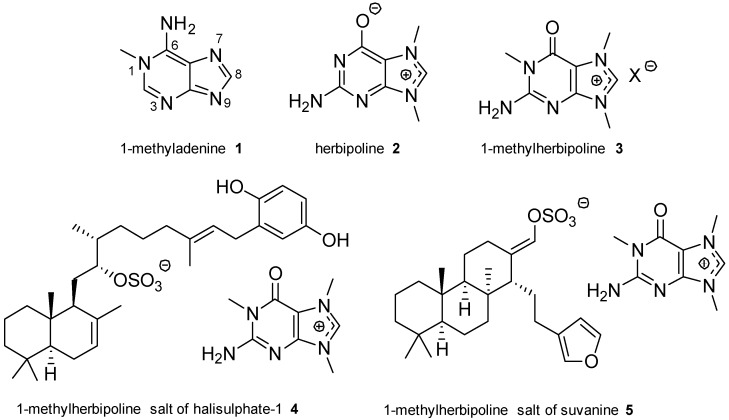
Structures of 1-methyladenine, herbipoline and related compounds.

Since the initial isolation of 1-methyladenine, **1**, and herbipoline, **2** (Figure 1), from the sponge *Geodia gigas* by Ackermann et al. in the early 1960s [5,6], a few more examples of methyl-guanines have been isolated from marine organisms.

From *Jaspis* sp. sponges collected off Hachijo-jima, Japan, Yagi et al. isolated 1-methylherbipoline, **3**, as a salt of an unidentified anion that showed a moderate collagenase inhibitory activity [7]. A few years later, Kimura et al. identified 1-methylherbipoline salts of halisulphate-1, **4**, and suvanine, **5**, from the sponge *Coscinoderma mathewsi* collected at Pohnpei, Micronesia (Figure 1). Both salts showed moderate antithrombin and antitrypsin activity, which was ascribed to the sesterterpene sulphate counterions rather than to the methylated purine [8]. 1-Methylherbipoline, **3**, was also isolated from a Papua New Guinea ascidian *Eudistoma* sp. [9].

**Figure 2:**
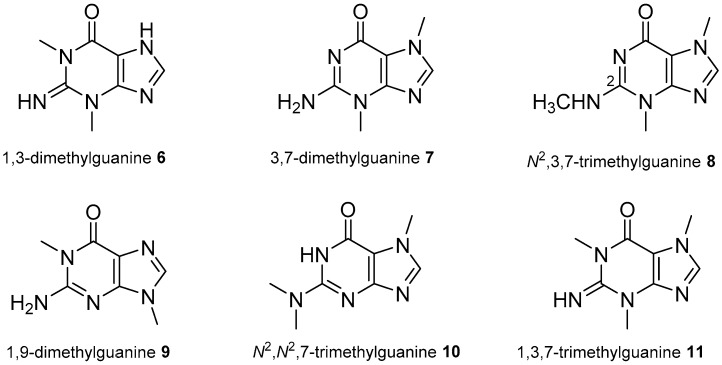
Structure of several methylated guanines.

1,3-Dimethylguanine, **6** (Figure 2), was isolated from the marine ascidian *Botrylloides leachi* in relatively high proportion, although its physiological role in ascidian was not determined. This marine organism was collected at Marlborough Sound, New Zealand [10].

3,7-Dimethylguanine, **7**, was isolated from the marine sponge *Zyzzya fuliginosa* collected in Batanes, Philippines. It was evaluated for cytotoxic activity, although it did not demonstrated bioactivity on the two cell lines tested, human T-cell leukemia CCRF CEM and human colon carcinoma HCT-116 [11]. A derivative of 3,7-dimethylguanine with an additional methyl at *N*^2^, i.e., *N*^2^,3,7-trimethylguanine, **8**, was isolated from the marine sponge *Petrosia* sp. collected at Jeddah, Saudi Arabia, on the Red Sea coast. It showed moderate to low cytotoxicity against human hepatocellular liver carcinoma (HepG2) and human breast adenocarcinoma (MCF-7) [12]. 1,9-Dimethylguanine, **9**, previously known as a synthetic compound, was isolated as a natural product from the Antarctic sponge *Isodictya erinacea* together with 7-methyladenine [13]. The trimethyl derivative *N*^2^,*N*^2^,7-trimethylguanine, **10**, has been identified from the New Zealand ascidian *Lissoclinum notti* [14].

1,3,7-Trimethylguanine, **11** (Figure 2), was first isolated from the sponge *Latrunculia brevis*, collected at the Otago Peninsula, New Zealand [15], and also detected in the methanol extract of an Indonesian ascidian *Eudistoma* sp. [16] and later from the Australian *Eudistoma maculosum* [17]. It was inactive against the P-388 murine leukaemia cell line in vitro (EC_50_ > 10 μg/mL) and had no detectable antiviral or antimicrobial activity.

### 2.2. Isoguanine Derivatives

**Figure 3:**
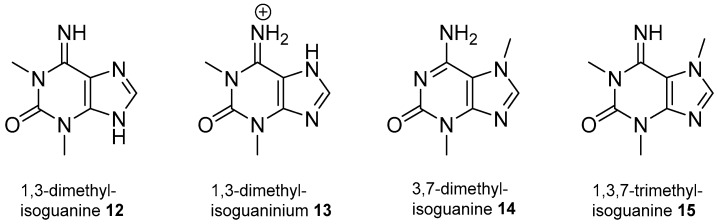
Structure of several methylated isoguanines.

Mitchell et al. [18] and Chehade et al. [19] reported almost simultaneously the isolation of 1,3-dimethylisoguanine, **12** (Figure 3), as a natural product from the same sponge species *Amphimedon viridis* (formerly known as *Haliclona viridis*), collected in Harrington Sound, Bermuda, and in the Sao Sebastiao channel, on the southeastern coast of Brazil, respectively. Its hydrochloric salt, in the form of 1,3-dimethylisoguaninium, **13**, was also reported from the sponge *Amphimedon paraviridis*, collected off Okinawa, Japan [20]. The same 1,3-dimethylisoguanine, **12**, was isolated from the New Zealand ascidians *Cnemidocarpa bicornuta* [21] and *Pseudodistoma cereum* [22]. It was tested on 26 human cancer cell lines showing the highest cytotoxicity on an ovarian cancer cell line (IC_50_ 2.1 μg/mL) [18]. Additionally, it was essentially inactive in haemolytic and antimitotic bioassays but stimulated mammalian gut motility in a dose-dependent manner [19] and showed a promising antiangiogenic activity [20].

3,7-Dimethylisoguanine, **14** (Figure 3), has been isolated from several marine organisms such as the sponges *Agelas longissima* [23] from the Caribbean Sea, *Zyzzya fuliginosa* (named as *Z. fuliginosus*) collected at Chuuk Atoll, Micronesia [24] and the gorgonian *Paramuricea clavata* [25], collected in the Western Mediterranean Sea. From the latter, the trimethylated derivative 1,3,7-trimethylisoguanine, **15**, was also isolated showing a potential antifouling activity with low toxicity [25]. The same 1,3,7-trimethylisoguanine, **15**, was previously isolated, for the first time as a natural product, from the New Zealand ascidian *Pseudodistoma cereum* [22].

**Figure 4:**
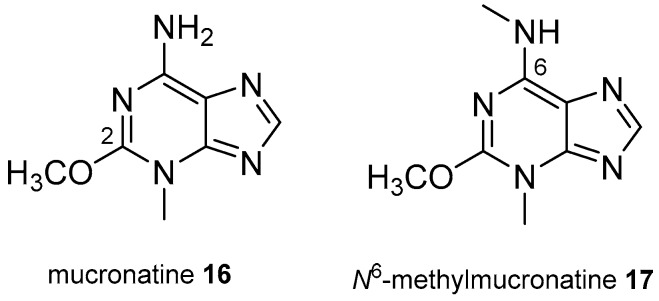
Structure of mucronatine, **16**, and methylmucronatine, **17**.

Purines methylated at the C2 or C6 groups have also been described as natural products. Thus, mucronatine, **16** (Figure 4), a rare example of purine with a methoxy group at C2, was identified from the sponge *Stryphnus mucronatus* collected in the Mediterranean Sea at La Ciotat (France) [26]. Mucronatine, **16**, can be considered as an *O*-methylisoguanine derivative that showed no cytotoxicity on KB cells, weak toxicity on the brine shrimp assay and promising antifouling properties. The trimethyl derivative *N*^6^-methylmucronatine, **17** (Figure 4), was more recently isolated from an unidentified dictyoceratid sponge collected in South East Queensland, Australia, and its structure was confirmed by X-ray crystallography [27].

**Figure 5:**
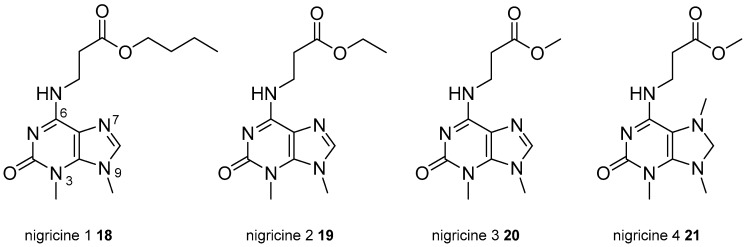
Structure of nigricines **18**–**21**.

Among the examples of methylisoguanines alkylated at the C6-amino group are nigricines 1–4, **18**–**21** (Figure 5), isolated from the sponge *Petrosia nigricans* collected in Pulau Baranglompo, Indonesia [28]. Nigricines 1–3 are 3,9-dimethylpurines, while nigricine 4 is a 3,7,9-trimethylated derivative. All of them bear a propanoate moiety at *N^6^* position and were not cytotoxic against the murine lymphoma cell line L5178Y.

### 2.3. Xanthine Derivatives

**Figure 6:**
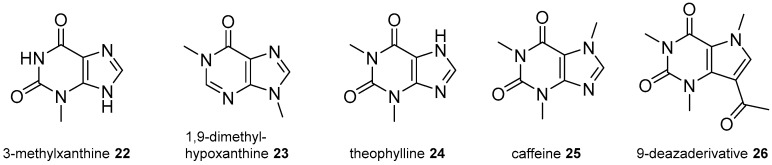
Structure of methylated xanthines **22**–**26**.

Few examples of xanthine derivatives have been identified from marine organisms. 3-Methylxanthine, **22** (Figure 6), was isolated from the ascidian *Symplegma rubra*, collected on the southeastern coast of Brazil [29]. Other analogues are 1,9-dimethylhypoxanthine, **23**, isolated from a southern Australian sponge *Spongosorites* sp. [30], theophylline, **24**, from *Amphimedon viridis* [18] and caffeine, **25**, from the gorgonian coral *Verrucella umbraculum* collected off the coast of Hainan Island, South China Sea, along with the 9-deazaderivative **26** [31].

### 2.4. 8-Oxopurine Derivatives

8-Oxopurines are uncommon in nature, although several analogues, derived from adenine, guanine or isoguanine, and bearing methyl substituents at various positions, have been isolated from marine organisms. Although they could have been considered in the previous sections as derivatives of the more common nucleic bases, we decided to group them in this section because of the unusual 8-oxo function.

**Figure 7:**
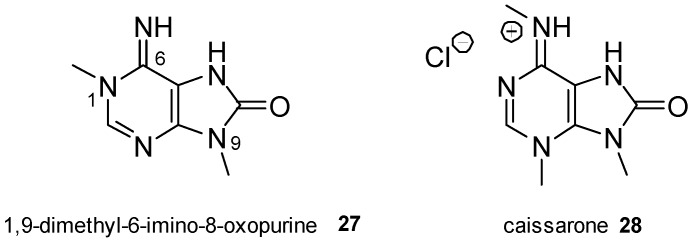
Structure of methylated 8-oxopurines **27** and **28**.

Thus, 1,9-dimethyl-6-imino-8-oxopurine, **27** (Figure 7), was isolated from the English Channel sponge *Hymeniacidon sanguinea* [32]. The unusual methylimino group at C6 is also present in caissarone, **28** (3,9-dimethyl-6-methylimino-8-oxopurine, Figure 7), which is a quaternary derivative isolated as the hydrochloride salt from *Bunodosoma caissarum*, a sea anemone abundant in the reefs of the Brazilian coast. This structure was confirmed by X-ray crystallography [33] and later by synthesis [34] (see Section 5). Caissarone was inactive against the P-388 leukaemia cell line [33], although it induced morphological anomalies in sea-urchin egg development [35] and acted as a simple competitive adenosine receptor antagonist [36].

**Figure 8:**
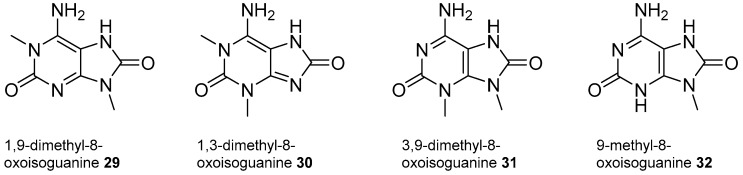
Structure of methylated 8-oxopurines **29**–**32**.

From the New Zealand ascidian *Pseudodistoma cereum* [37] collected at the Three Kings Islands, Appleton et al. isolated an 8-oxopurine derivative that had no detectable cytotoxic, antibacterial or antifungal activities. They identified it as 1,3-dimethyl-8-oxoisoguanine, although this structure was corrected a few years later by Sakaruda et al., who isolated a series of methylated isomers of 8-oxoisoguanines from several Palauan sponges including *Cribrochalina olemda*, *Haliclona* sp. and *Amphimedon* sp. [38]. These isomers were identified as 1,9-dimethyl-8-oxoisoguanine, **29**, 1,3-dimethyl-8-oxoisoguanine, **30**, 3,9-dimethyl-8-oxoisoguanine, **31**, and 9-methyl-8-oxoisoguanine, **32** (Figure 8). The thorough spectroscopic analysis of these compounds performed by Sakurada et al. suggested that the isoguanine previously isolated from the ascidian *P. cereum* suited better the 3,9-isomer rather than the 1,3-isomer proposed by Appleton [37]. These 8-oxoisoguanines seemed to be responsible for the convulsant effect observed for the aqueous extracts of these Palauan sponges, being potential new neuropharmacological agents. Indeed, 1,9-dimethyl-8-oxoisoguanine, **29**, induced convulsions in a dose-dependent manner through inhibition of neuronal GABAergic transmission [38].

**Figure 9:**
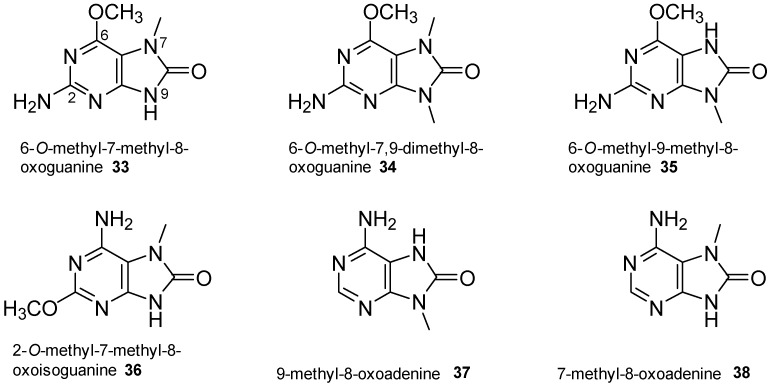
Structure of methylated 8-oxopurines **33**–**38**.

Other natural 8-oxopurines were isolated from two specimens of the ascidian *Symplegma rubra* collected on distant geographical areas. 6-*O*-Methyl-7-methyl-8-oxoguanine, **33** (Figure 9), was first isolated from *S. rubra* collected on the southeastern coast of Brazil [29] along with 8-oxoadenine and 3-methylxanthine, **22**. The same three purines were identified from a specimen of *S. rubra* collected at the Saudi Red Sea coast [39] along with several other unknown and known purine derivatives (Figure 9): 6-*O*-methyl-7,9-dimethyl-8-oxoguanine, **34**, 6-*O*-methyl-9-methyl-8-oxoguanine, **35**, 2-*O*-methyl-7-methyl-8-oxoisoguanine, **36**, 9-methyl-8-oxoadenine, **37**, 7-methyl-8-oxoadenine, **38**, and the nucleoside inosine. These 8-oxopurines showed moderate inhibitory activity against several protein kinases [39].

## 3. *N*-Alkylpurines with Substituents Other Than Methyl

Apart from the methylpurines shown above, derivatives with larger alkyl chains have been isolated from marine organisms. Perhaps the most common compounds are those bearing terpenic chains attached to adenine residues, for which a variety of biological properties have been described and covered by previous reviews [40,41]. However, in this section, we have decided to include all the terpenylpurines described, but grouped according the terpenoid skeleton of the chain instead the usual chronological isolation sequence, with the aim to give a more intuitive insight into the structures.

### 3.1. Terpenylpurines

**Figure 10:**
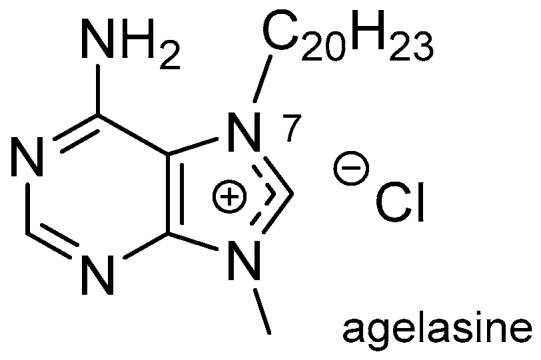
Structure of agelasine.

Most of the compounds included in this group are derived from 9-methyladenine, mainly as an adeninium salt. The first compound of this type was described in 1975 by Cullen et al. [42], who described the isolation of a secondary metabolite from the sponge *Agelas dispar*, collected at Barbados that was called agelasine (Figure 10), in which a terpenyl moiety was attached to position 7 of a quaternary 9-methyladeninium.

The structure of the terpenyl core was undefined although it was described as a bicyclic diterpene. Since then, many other diterpenylpurines have been isolated from different marine organisms. They are derived either from adenine or from other purines with diterpenyl groups of different nature, including acyclic, monocyclic and bicyclic terpenyl moieties. They are described below starting from the acyclic diterpenes.

#### 3.1.1. Terpenylpurines with an Acyclic Diterpenoid

**Figure 11:**
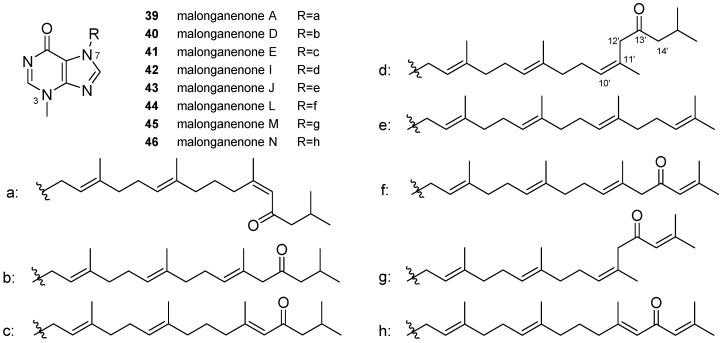
Structure of malonganenones **39**–**46**.

**Figure 12:**
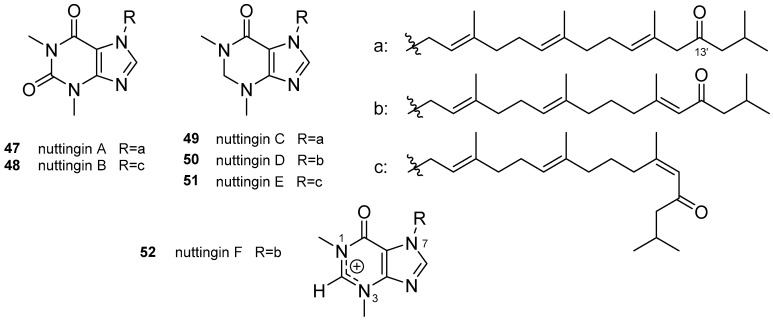
Structures of nuttingins **47**–**52**.

There are two groups of marine metabolites, named malonganenones (Figure 11) and nuttingins (Figure 12), that bear an acyclic diterpenoid geranylgeranyl chain attached to the position 7 of purine heterocycles such as 3-methylhypoxanthine, 1,3-dimethylxanthine and 1,3-dimethylhypoxanthine.

Malonganenones (Figure 11) are isolated from gorgonians. Malonganenone A, **39**, was first isolated from the gorgonian *Leptogorgia gilchristi*, collected near Ponto Malongane, Mozambique [43]. It was also isolated together with the new derivatives malonganenones D, **40**, and E, **41**, from the gorgonian *Euplexaura nuttingi* collected in Pemba Island, Tanzania [44]. Other compounds of the same family are malonganenones I, **42**, and J, **43**, identified from the South China Sea gorgonian *Euplexaura robusta* collected on Weizhou Island, China [45]. The last compounds identified of this group are malonganenones L, **44**, M, **45**, and N, **46** (Figure 11), isolated from the gorgonian *Echinogorgia pseudossapo* collected in Daya Bay, China [46]. All of them are 3-methylhypoxanthines with the diterpenyl chain attached to position 7. Malonganenone J, **43**, has an unmodified geranylgeranyl side chain, while the others have a carbonyl function at C13′ and differ in the presence or absence of a double bond at C14′ and in the position and Z–E configuration of the double bond at C10′ or C11′. These compounds were evaluated for their cytotoxicity against K562 and HeLa tumour cell lines and it was found that malonganenones A, D, E and I, **39**–**42**, showed good to moderate cytotoxicities with IC_50_ values ranging from 0.35 to 10.82 μM while malonganenone J, **43**, showed IC_50_ > 53 μM, suggesting that the carbonyl function at the side chain was important for the bioactivity. The inhibitory activity against the receptor tyrosinekinase (RTK) c-Met was also evaluated but only malonganenone E, **41**, showed moderate inhibitory activity at a concentration of 10 μM [45]. Malonganenones L–N, **44**–**46**, were also tested as inhibitors of several phosphodiesterases showing moderate activity [46].

Others secondary metabolites isolated from these gorgonians are also named malonganenones and have the same tetraprenylated side chains but they are not purine derivatives. Malonganenones B, F, G, O, P and Q have the diterpenic chain joined to a trisubstituted imidazole fragment and could be biosynthetic precursors of the above purines and malonganenones C, H and K are *N*-diterpenylformamides [43,44,45,46].

During the same study on the Tanzanian gorgonian *E. nuttingi*, nuttingins A–F, **47**–**52** (Figure 12), were also isolated and identified having the same 13′-oxo-prenylated side chains of malonganenones. Nuttingins A and B, **47**–**48**, are 1,3-dimethylxanthine derivatives; nutingins C–E, **49**–**51**, are 1,3-dimethyl-2,3-dihydrohypoxanthines and nuttingin F, **52**, is a 1,3-dimethylhypoxanthine derivative with an unusual naturally positive charge delocalised over the two nitrogens N1 and N3. Nuttingins A–E, **47**–**51**, induce apoptosis in transformed mammalian cells and displayed inhibitory activity against both K562 and UT7 tumour cell lines; nuttingins C–E, **49**–**51**, are approximately three-fold more potent than the others [44].

#### 3.1.2. Terpenylpurines with a Monocyclic Diterpenoid

**Figure 13:**
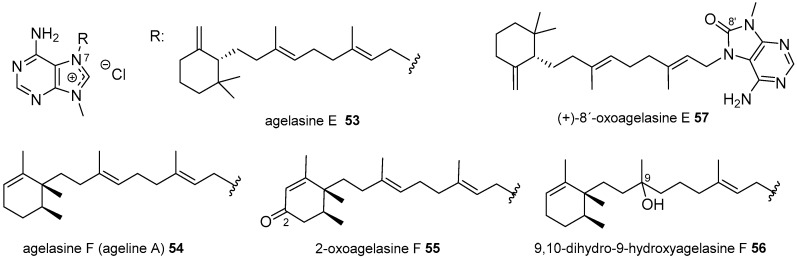
Structure of agelasines E and F and related compounds **53**–**57**.

Capon and Faulkner [47] isolated a 9-methyladeninium salt that they called ageline A (Figure 13), from *Agelas* sp. collected at Palau, Western Caroline Islands, for which mild ichthyotoxic and moderate antimicrobial activities were described and which contain a monocyclic diterpenoid fragment. Almost simultaneously, Nakamura et al. [48] isolated another two 9-methyladeninium salts with a monocyclic diterpenoid chain from the Okinawan Sea sponge *Agelas nakamurai*, named agelasine E and F, **53**–**54** (Figure 13). Agelasine F, **54**, was proven to be identical to ageline A; since then, all the diterpenoid 9-adeninuim salts are named agelasines. Agelasine F, **54**, has also been isolated from an orange *Agelas* sp. collected at Baler, Philippines [49] and from *Agelas axifera* from Republic of Palau [50]. Both showed interesting antituberculosis activity [49,51]. 

Recent studies allowed the isolation of new analogues oxidised at the terpenic chain, i.e., 2-oxoagelasine F, **55**, and 9,10-dihydro-9-hydroxyagelasine F, **56**, from *A. nakamurai* [52], and oxidised at the purine as (+)-8′-oxoagelasine E, **57**, (Figure 13), from the South China Sea sponge *Agelas mauritiana* [53].

#### 3.1.3. Terpenylpurines with a Bicyclic Diterpenoid

In this section, the largest group of terpenyladenine marine metabolites are considered. They are again derived mainly from 9-methyladeninium and the diterpenoid chain attached to position 7 can be considered to be derived from labdane, clerodane and halimane type skeletons, which can be found in both enantiomeric series or epimerised at any position with a *trans*- or *cis*-fused decaline structures. Halimanes and clerodanes arise biogenetically from labdanes by one and two methyl migrations, respectively, and, sometimes, the three types are found in the same species. Although the agelasines have been given correlative letters from A to V independently of the kind of diterpene skeleton, we have grouped them according to the diterpenoid type rather than following a chronological description to better underscore the structural similarity. The relationship between the structure of the diterpene skeleton and the letter assigned to each compound is shown in Table 1.

##### Agelasines with a Clerodane Type Diterpenoid

**Figure 14:**
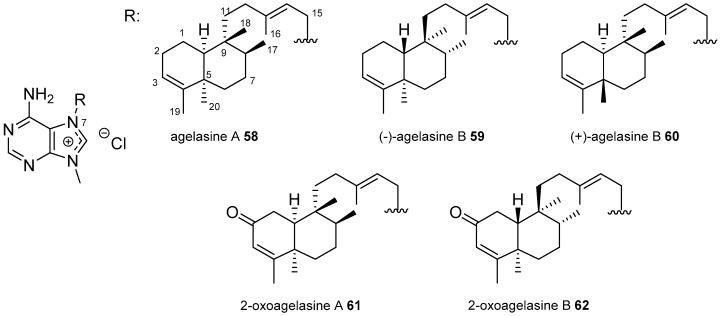
Structure of agelasines A and B and analogues **58**–**62**.

The first agelasines with a clerodane type diterpenoid were agelasine A, **58**, and agelasine B, **59** (Figure 14), isolated by Nakamura et al. from *A. nakamurai* collected at Zanpa Cape, Okinawa [54,55]. Both compounds differ in the stereochemistry of the decaline core. The enantiomer (+)-agelasine B, **60**, has also been isolated from *A. mauritiana* collected at Yongxing Island in the South China Sea [53]. The corresponding analogues with a carbonyl group at position 2 of the diterpenoid moiety, 2-oxoagelasine A, **61**, and 2-oxoagelasine B, **62**, were isolated several years later from the same species collected at Okinawa [52] and an *Agelas* sp. from Papua New Guinea, respectively [56].

**Figure 15:**
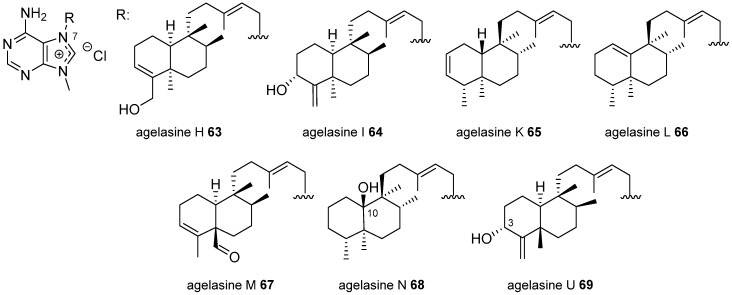
Structures of agelasines **63**–**69**.

The isomers agelasine H, **63**, and agelasine I, **64**, (Figure 15), were isolated from another *Agelas* sp. collected at Yap Island, Micronesia and they differed in the position of an allylic hydroxyl group [57]. They were isolated together with agelasines A, **58**, agelasine B, **59**, and agelasine F, **54**, and some of them showed varied degrees of antimicrobial activity.

The next clerodane type derivatives are agelasine K, **65**, and agelasine L, **66**, isolated from *A*. *cf. mauritiana* collected at Solomon Islands [58], which differ in the position of a double bond on the decaline core (Figure 15). They showed mild activity on *Plasmodium falciparum*. Agelasine M, **67**, and 2-oxoagelasine B, **62**, identified from a Papua New Guinea *Agelas* sp. showed interesting activity against *Trypanosoma brucei* [56]. A new derivative with a hydroxyl group at position 10, agelasine N, **68**, was isolated from the Caribbean sponge *Agelas citrina* together with some previously described agelasines [59]. Agelasine U, **69**, isolated from an Okinawan *Agelas* sp. and belonging to the enantiomeric series as **67**, had also a hydroxyl group but at C3 [60].

**Figure 16:**
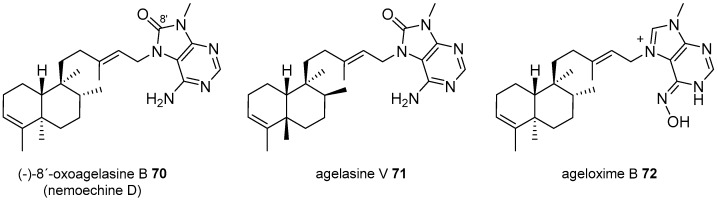
Structures of agelasines **70**–**72**.

Derivatives with different purine moieties have been identified from *A. mauritiana* collected at different places, such as the 8-oxopurine analogues (−)-8′-oxoagelasine B, **70**, and agelasine V, **71**, (Figure 16), from the sponge collected at Yongxing Island [53] and the oxime derivative, ageloxime B, **72**, from a specimen collected at Paracel Islands [61], both in the South China Sea. Antibacterial and cytotoxic assays, performed with these compounds, seem to indicate that those with the 8′-oxo function were less potent than the other analogues [53]. The agelasine **70** was almost simultaneously isolated from *Agelas* aff. *nemoechinata* collected also at Paracel Islands but it was given the name nemoechine D [62].

**Figure 17:**
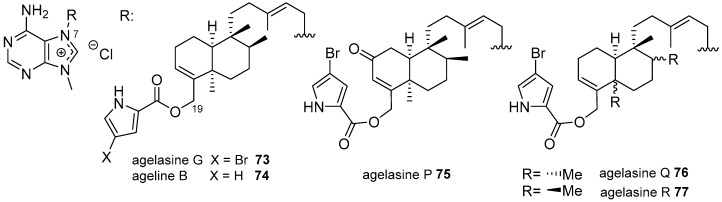
Structure of agelasines **73**–**77**.

There are some clerodane derivatives that also bear a pyrrole or bromopyrrole attached to position 19 of the diterpenoid by an ester function. The first of this group were agelasine G, **73**, and ageline B, **74**, (Figure 17), isolated first from an Okinawan *Agelas* sp. [63] and later on from *A. nakamurai* [49,52]. In addition to anti-leukaemic activity, agelasine G showed selective inhibition of protein tyrosine phosphatase 1B, which suggest activation of the insulin signalling pathway, while the analogue lacking bromine was nearly inactive [64]. Other agelasines with a bromopyrrole substituent on a clerodane skeleton are agelasines P, Q and R, **75**–**77** (Figure 17) [60].

##### Agelasines with an Halimane Type Diterpenoid

**Figure 18:**
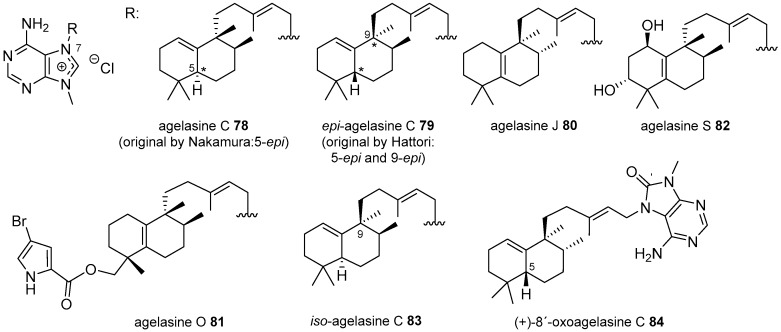
Structures of agelasines **78**–**84**.

The first described 9-methyladeninium alkylpurines with an halimane derivative were agelasine C, **78**, (Figure 18), from the Okinawan *Agelas* sp. [54] and later from *A. mauritiana* collected from Pohnpei, Micronesia, together with epi-agelasine C, **79**, for which antifouling activity against macroalgae was described [65]. The structure of these compounds was later corrected to that shown in Figure 18 after the synthesis of its enantiomer by Marcos et al. [66].

Agelasine J, **80**, was isolated from *A.* cf*. mauritiana* [58] together with the clerodane agelasines K and L, **65**–**66**. Other examples isolated from the Okinawan *Agelas* sp., are agelasines O and S, **81**–**82**, with a bromopyrrole substituent and two hydroxyl groups at the decaline core, respectively [60].

The sponge *A. nakamurai*, collected at Xisha Islands in the South China Sea, yielded iso-agelasine C, **83**, (corresponding to 9-epi-agelasine C) together with agelasines B, **59**, J, **80**, and 2-oxo-agelasine B, **62**, for which cytotoxic, antifungal and antibacterial activities were evaluated [67]. It was found that the presence of a carbonyl in the diterpene skeleton decreases cytotoxic and antimicrobial activities. In this group, an 8-oxopurine derivative has also been recently described, namely 8′-oxoagelasine C, **84** (Figure 18) [53]. 

**Figure 19:**
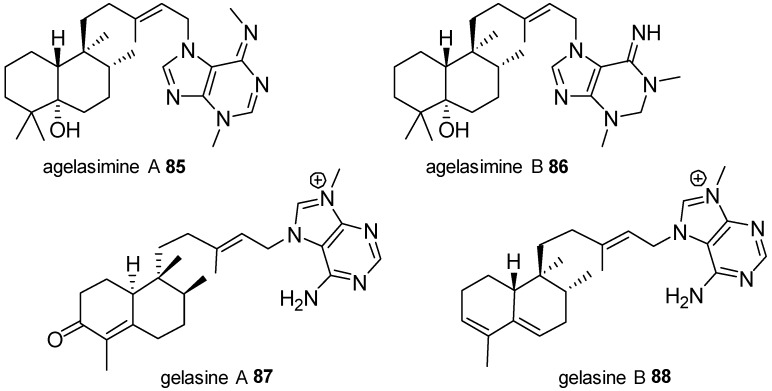
Structures of agelasimines A and B and gelasines A and B.

Derived from an halimane diterpenoid are the only two non-quaternary adenine derivatives, agelasimine A, **85**, and B, **86**, (Figure 19), that were isolated from *A. mauritiana* collected at Enewetak Atoll, Marshall Islands (Pacific Ocean) [68]. Both have the same diterpenic moiety attached to position N7 of 3,*N*^6^-dimethyladenine and 1,3-dimethyladenine, respectively. They have been tested for a variety of biological activities and both were cytotoxic against L1210 leukaemia cells at nanomolar range. They also inhibited smooth muscle contractions induced by potassium chloride and reversed the effects of a variety of neurotransmitters at micromolar concentrations. It was proposed that may act as Ca^2+^-channel antagonists and α_1_ adrenergic blockers [69].

Two unusual nor-diterpenoid derivatives, gelasine A and B, **87**–**88** (Figure 19), were isolated from the Papua New Guinea *Agelas* sp. in which the C19 methyl group is lost [56]. Gelasine A, **87**, has an α,β-unsaturated carbonyl, and gelasine B, **88**, a conjugate diene with an enantiomeric decalin core.

##### Agelasines with a Labdane Type Diterpenoid

Finally, labdane type agelasines have also been isolated from genus *Agelas*. Agelasine D is one of the examples in which both enantiomers have been described from different specimens of the same species. (+)-Agelasine D, **89**, was described by Nakamura in 1984 together with agelasines A–C, **58**, **59** and **78**, from *A. nakamurai* showing inhibitory effects on Na^+^,K^+^-ATPase as well as antimicrobial activity and an interesting cytotoxicity against several cell lines [54,55].

**Figure 20:**
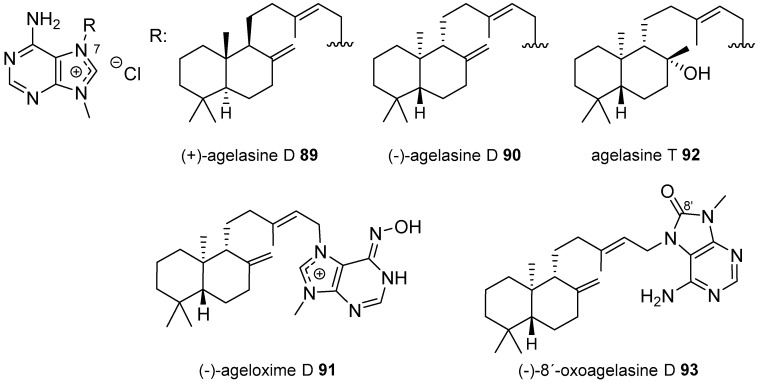
Structures of agelasines **89**–**93**.

The enantiomer (−)-agelasine D, **90**, and its oxime derivative (−)-ageloxime D, **91**, (Figure 20) were also identified from a specimen of *A. nakamurai* collected at Bali Island, Indonesia for which not only cytotoxicity has been described but also anti-fouling activity [70]. The results indicate that (−)-agelasine D, **90**, was more potent as a cytotoxic agent and the oxime derivative showed better results on the anti-fouling assay. They were tested also for biofilm inhibition against *Staphylococcus epidermidis*, founding that (−)-agelasine D, **90**, inhibits the growth of the planktonic bacteria but not the biofilm formation, while the oxime had the opposite behaviour [70]. A hydroxylated labdane is present in agelasine T, **92** (Figure 20), isolated from an Okinawan *Agelas* sp. that showed antimicrobial activity against several bacteria and fungi [60]. (−)-8′-Oxoagelasine D, **93**, was identified from two species of the Paracel islands, *A. mauritiana* [61] and *A.* aff. *nemoechinata* [62]. It showed antifungal and anti-leishmanial activity as did the other oxime derivatives (−)-ageloxime B, **72**, and (−)-ageloxime D, **91**, isolated in one of the studies [61], as well as cytotoxicity against the HeLa cell line [62].

(−)-8′-Oxoagelasine D, **93**, and (+)-agelasine D, **89**, were also identified, from *A. mauritiana* collected at Yongxing Island in the South China Sea together with other 8′-oxo derivatives described above with clerodane and halimane diterpenoids [53]. Antibacterial and cytotoxic assays seem to indicate that compounds with the 8′-oxo function were less potent than the other analogues.

##### Diterpenylpurines Other Than Agelasines

**Figure 21:**
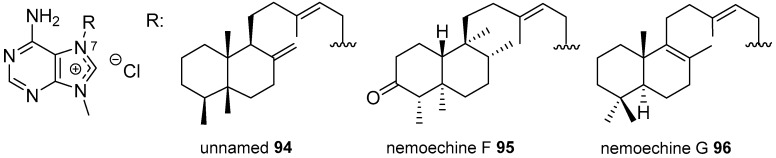
Structure of terpenylpurines **94**–**96**.

The unnamed diterpenylpurine **94** (Figure 21), having an unusual telepogane skeleton which could be considered in the midway from labdane to clerodane, was isolated from *A. nakamurai* from Fly Islands, Papua New Guinea, together with agelasine B, **59** [71].

From *A.* aff. *nemoechinata* collected at the coral reef regions in the South China Sea, two new diterpene derivatives, nemoechine F and G, **95**–**96**, were isolated (Figure 21). They have clerodane and labdane skeletons respectively. They were tested for antimicrobial and cytotoxic activities against several tumour cell lines but only nemoechine G, **96**, showed weak cytotoxicity against Jurkat cell line [72]. Nemoechine G, **96**, was also identified in the South China Sea *A. nakamurai* [67].

#### 3.1.4. Terpenylpurines with a Diazepinopurine Heterocyclic System

Among the terpenylpurines with a bicyclic diterpenoid, particular attention has to be given to the asmarines, a group of 7-terpenylpurines isolated from sponges of genus *Raspailia*, in which the amino group of the adenine group is cyclised over the side chain of a clerodane diterpenoid forming a new diazepinopurine system. Unlike the agelasines, all of them are neutral and the differences among them lie in the oxidation degree of the heterocyclic ring and in the ring junction of the decalin core.

**Figure 22:**
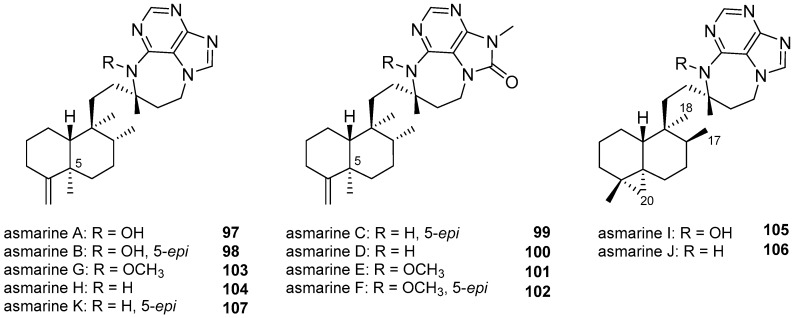
Structures of asmarines **97**–**107**.

In 1998, Kashman et al. described the first members of this group, asmarines A and B, **97**–**98** (Figure 22), as the major components of a Red Sea *Raspailia* sp. collected at Nakora, Eritrea [73]. They had a hydroxyl substituent on the diazepine nitrogen and are epimers at position 5. Two years later, and from the same sponge, four additional asmarines C–F, **99**–**102** (Figure 22), were isolated as two inseparable pairs of compounds that derived from 9-methyl-8-oxopurine and differ also in the cis/trans junction of the decalin [74]. Asmarines C and D, **99**–**100**, have no substitution on the nitrogen of the diazepine and asmarines E and F, **101**–**102**, had a methoxy group at that position.

From a different *Raspailia* sp., collected at Wasini Island, Kenya, asmarines G and H, **103**–**104**, were identified together with asmarines A, **97**, and F, **102**, the latter being the major component (Figure 22) [75]. Similar results were obtained with a *Raspailia* sp. collected at Nosy Be, Madagascar, from which asmarines A, **97**, and F, **102**, were the major components but three new asmarines I, J and K, **105**–**107**, were also identified: asmarine K, **107**, is the 5-*epi* isomer of asmarine H, **104**, and asmarines I and J, **105**–**106**, have the same diterpenoid moiety differing in the presence of a hydroxyl group in the nitrogen of the diazepine ring [76]; this diterpenoid moiety has the methyl C20 being part of a cyclopropane ring and also the methyl groups C17 and C18 are in trans disposition instead the cis one found in the rest of asmarines.

These compounds have proved to be cytotoxic against several tumour cell lines with asmarine B, **98**, being the most potent on human lung and colon carcinomas [73,75]. Not all the asmarines were evaluated because of the small amounts isolated.

Several analogues in which the decalin core has been substituted by simple chains have been synthesised and found that they can also been even more potent as cytotoxics than the natural model compounds [77]. The work of Shenvi and other authors such as Kashman, Gundersen and Ohba on asmarine analogues will be discussed in Section 5.

### 3.2. Other Alkylpurines

In this section, we consider those alkylpurines with substituents other than methyl or diterpenyl substituents at any nitrogen of the purine system.

**Figure 23:**
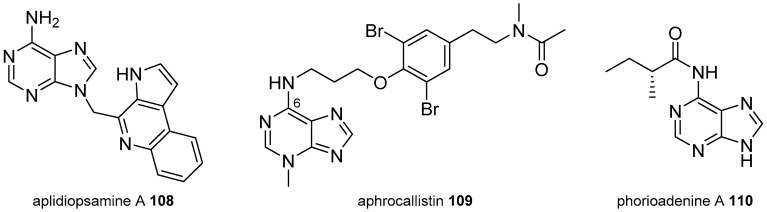
Structures of alkylpurines **108**–**110**.

Several adenines are included in this section, such as the unique adenine derivative with a tricyclic aromatic substituent, aplidiopsamine A, **108**, (Figure 23), which was isolated from the Australian ascidian *Aplidiopsis confluata* collected from Tasmania and showed interesting anti-plasmodial activity on chloroquine-resistant and chloroquine-sensitive strains of *Plasmodium falciparum* with low toxicity towards human cells [78].

Other adenine and oxoadenine derivatives are alkylated at the *N*^6^ amino group. Aphrocallistin, **109** (Figure 23), is a 3-methyladenine bearing a bromotyrosine-derived substituent at *N*^6^ having a naturally uncommon dialkylamide; it was isolated from the sponge *Aphrocallistes beatrix*, collected at the Coral Reefs of Florida, near Fort Pierce [79]. Its structure was confirmed by synthesis and the compound was evaluated in a panel of human tumour cell lines with significant selectivity on colon, melanoma and breast cancer cell lines [79]. Several other pharmacological assays were performed to determine its solubility, permeability or metabolic properties. It was also tested as antimicrobial, although no significant activity was detected [79].

An *N*^6^-acyladenine derivative, phorioadenine A, **110** (Figure 23), was identified from the Australian sponge *Phoriospongia* sp., showing a very interesting nematocidal activity but it was non-antibacterial and non-cytotoxic on several mammalian cancer cell lines [80]. The enantiomer, the racemic and three achiral analogues, obtained by synthesis, were inactive as anthelmintics, suggesting the importance of the nature and chirality of the *N*-acyl group [80].

**Figure 24:**
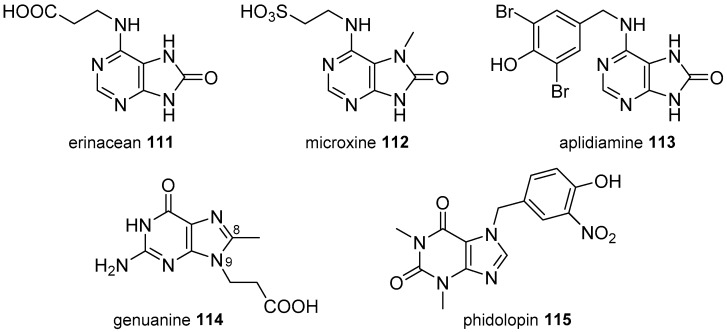
Structures of purines **111**–**115**.

From the sponge *Isodictya erinacea* from McMurdo Sound, Antarctica, erinacean **111** was identified as an 8-oxoadenine alkylated at *N*^6^, (Figure 24) [13]. It had weak antibiotic activity and moderate cytotoxicity. Other related 8-oxoadenine derivatives are microxine, **112**, isolated from the Australian sponge *Microxina* sp., which had a weak inhibitory cdc2 kinase activity [81] and aplidiamine, **113**, isolated from the ascidian *Aplidiopsis* sp. [82,83].

From the venom of two cone snail species, *Conus genuanus*, collected in Sao Vicente, Cape Verde and *Conus imperialis*, from Hawaii, an unusual small molecule derived from guanine, genuanine, **114**, was isolated (Figure 24). It has a methyl at C8 of the purine ring and a propionic acid at position 9 [84]. Its structure was confirmed by synthesis and some regioisomers were also obtained. It induced paralysis in mice at the nanomolar level mimicking the potent paralytic activity found in the venom extract [84].

Phidolopin, **115**, is a relatively rare natural xanthine containing a nitro group that was isolated from the bryozoan *Phidolopora pacifica*, collected off Vancouver Island, Canada. It is considered largely responsible for the antifungal and anti-algae activities of the extracts [85].

**Figure 25:**
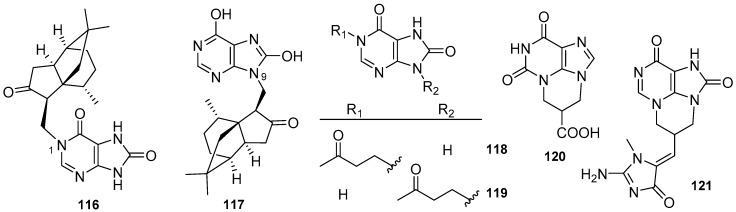
Structures of alkylpurines **116**–**121**.

From the South China Sea gorgonian *Subergorgia suberosa*, the dioxopurine/enol tautomer derivatives **116**–**121**, (Figure 25), were isolated. Two of them, **116** and **117**, have the sesquiterpenoid suberosanone as substituent either at 1 or 9 positions; the next two compounds, **118** and **119**, bear a 2-oxobutyl rest also at 1 or 9 positions; and derivatives **120** and **121** have an additional pyrimidine fused ring. All of them showed from moderate to weak cytotoxicity towards two human cancer cell lines [86,87,88]. 

**Figure 26:**
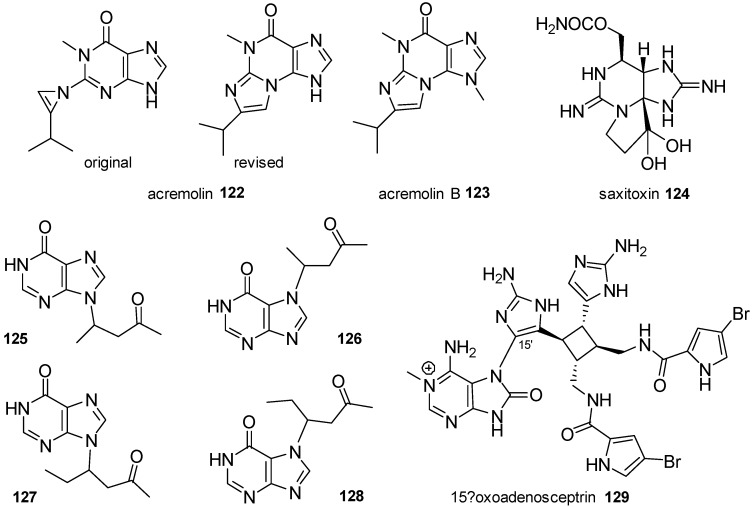
Structures of alkylpurines **122**–**129**.

Acremolin, **122**, was obtained in 2012 by fermentation of the marine-derived fungus *Acremonium strictum* [89], (Figure 26). Its structure was revised from a 1*H*-azirine substituted purine to an imidazopurine system showed in Figure 26; this revision was done initially by Banert [90] and confirmed a year later by Januar and Molinski, after its synthesis from guanosine [91]. The synthesis that corroborated the structure will be described later in this review. Recently acremolin B, **123**, has been isolated from another deep-sea derived fungus *Aspergillus* sp. from the Indian Ocean [92].

Several neurotoxins such as saxitoxin, **124** (Figure 26), and analogues, produced by cyanobacterias and certain marine dinoflagellates, include in their structures a perhydropurine system. They have been the subject of very good reviews, and thus they are not considered here [93,94,95].

Two pairs of alkylhypoxanthine regioisomers **125**–**126** and **127**–**128** were isolated from the sponge *Haliclona cymaeformis*, collected at Xuwen coral reef in the South China Sea [96]. They were tested for their cytotoxic, antibacterial and antifungal activities but they were found to be inactive (Figure 26). 

An atypical hybrid between an 8-oxoadenine and the pyrrole-imidazole alkaloid sceptrin, that was named 15′-oxoadenosceptrin, **129**, was identified from *Agelas sceptrum* from Plana Cays, Bahamas. It did not show cytotoxicity and was inactive as antimicrobial (Figure 26) [97].

## 4. Nucleoside Derivatives

**Figure 27:**
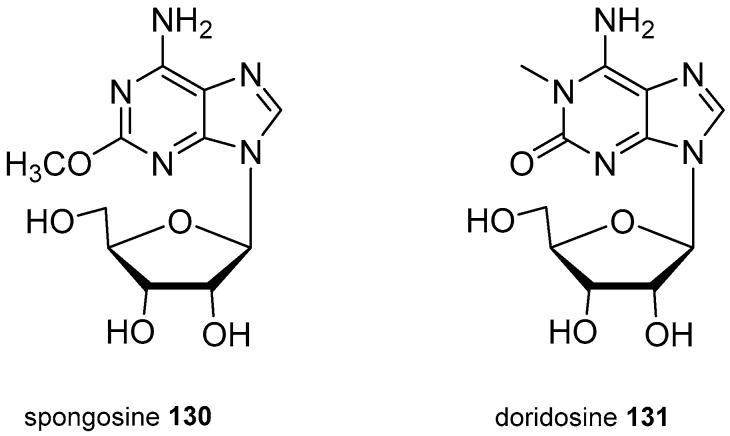
Structures of spongosine and doridosine.

Modified nucleosides are relatively uncommon secondary metabolites in marine organisms, although they are known since the early work performed by Bergmann during the 1950s on different Caribbean sponges. That work led to the identification, among others, of spongosine, **130** (2-methoxyadenosine, Figure 27), which may be the first purine nucleoside of marine origin isolated from the Caribbean sponge *Tectitethya crypta* (named as *Cryptotethya crypta*) [98]. Since then, several other purine, pyrimidine and deazapurine nucleosides have been described from different marine sources and collected in previous reviews [99,100]; thus, they are not considered in this one unless they have any unusual structural feature such as additional alkylated nitrogen, uncommon substitutions either on the sugar or the purine moieties, or interesting biological activities.

Doridosine, **131**, (1-methylisoguanosine, Figure 27) has been isolated from the Australian sponge *Tedania anhelans* (named as *T. digitata*) [101] and from the nudibranch *Peltodoris nobilis* (named as *Anisodoris nobilis*) [102,103]. It showed skeletal muscle relaxant, hypothermic and cardiovascular effects mediated via adenosine A_1_ and A_2_ receptors [101,103,104].

**Figure 28:**
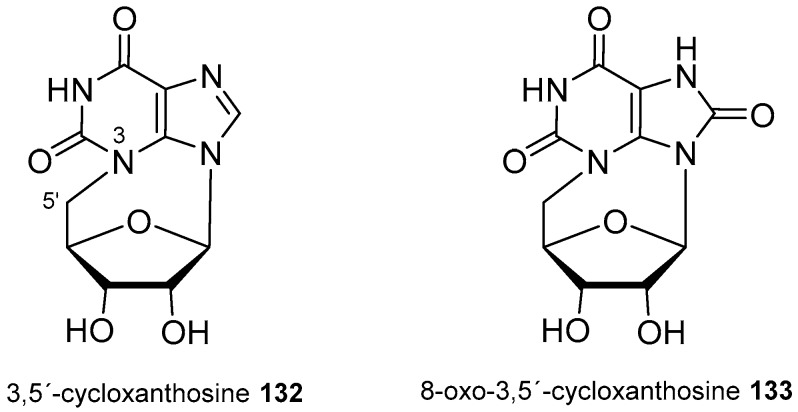
Structures of cyclonucleosides **132** and **133**.

Among cyclonucleosides, 3,5′-cycloxanthosine, **132** (Figure 28), was described by the first time as a natural compound from the Australian sponge *Eryus* sp. [105], and later on from the Mediterranean sponge *Axinella polypoides* collected in the Bay of Calvi, Corsica (France), together with a new cyclonucleoside identified as 8-oxo-3,5′-cycloxanthosine, **133** [106]. Some of these cyclonucleosides were known as synthetic compounds before they were identified as natural products [105,106,107].

**Figure 29:**
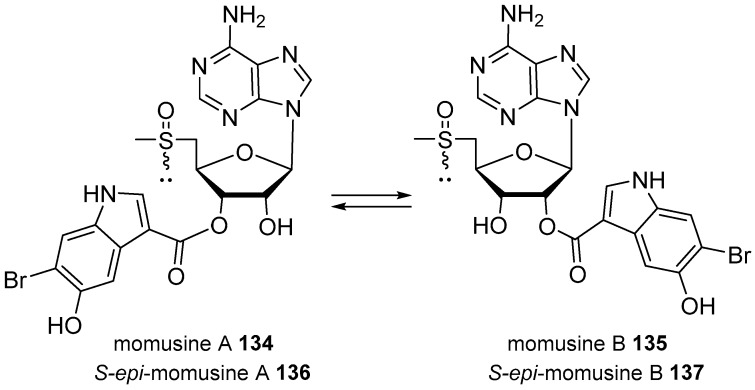
Structures of momusines **134**–**137**.

Four unusual methylsulphinyladenosine derivatives (Figure 29), momusine A/B, **134**/**135**, and epi-momusine A/B, **136**/**137**, were isolated from the ascidian *Herdmania momus* collected at the Korea coast; they are interconvertible transesterification isomers (ratio 56/44) and sulphinyl epimers bearing a 6-bromo-5-hydroxyindol-3-ylcarbonyl and a rare methylsulphinyl moieties on the sugar. These compounds were evaluated for antiviral activity against human pathogenic viruses but none of the isomeric mixtures showed significant antiviral effects [108].

**Figure 30:**
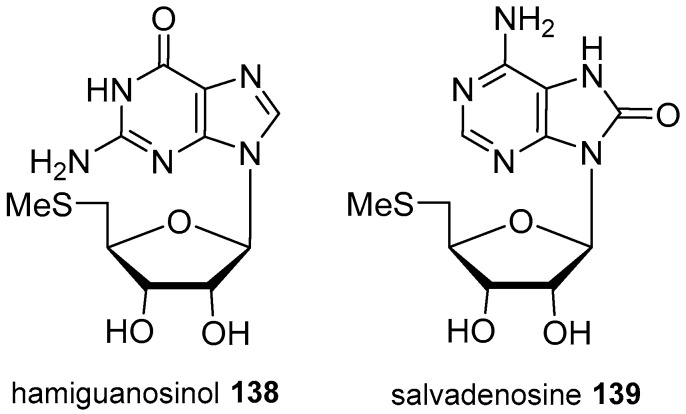
Structures of hamiguanosinol and salvadenosine.

From the Mediterranean sponge *Hamigera hamigera*, collected near Elba, Italy, a modified nucleoside with a 5′-deoxy-5′-methylthioribose moiety, named hamiguanosinol, **138**, was isolated (Figure 30) [109]. Initially, the enol form at C6 was proposed but it was later corrected to the more usual keto form by Jamiston et al. after the isolation of salvadenosine, **139**, a new 5′-methylthioribose nucleoside from the deep-water tunicate *Didemnum* sp. collected off Little San Salvador Island (Bahamas) [110].

**Figure 31:**
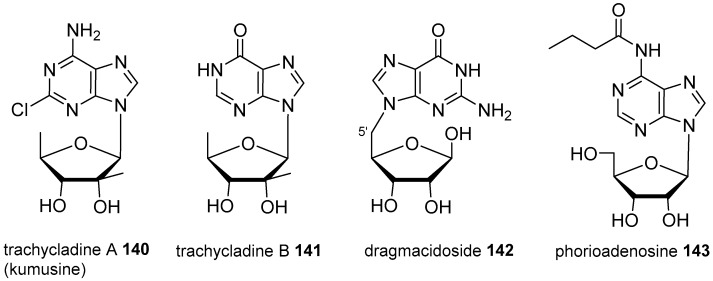
Structures of nucleosides **140**–**143**.

The rare chloroadenosine analogue trachycladine A, **140** (Figure 31), with a 5-deoxy-2-C-methyl sugar was identified from the sponge *Trachycladus laevispirulifer* from Exmouth Gulf (Australia) together with trachycladine B, **141**, the corresponding hypoxanthine nucleoside [111]. Trachycladine A showed cytotoxicity against several human cell lines but it was inactive against some yeasts and bacteria [111]. Simultaneously, the same adenosine nucleoside **140** was described from an Indonesian sponge, *Theonella* sp., and given the name kumusine [112] and later it was also identified in a specimen of *Theonella cupola* collected in Okinawa [113].

A recent example of new marine nucleoside, with a ribose linked to a guanine through C5′, is dragmacidoside, **142** (Figure 31), isolated from the sponge *Dragmacidon coccineum* (named as *D. coccinea*) collected from the Red Sea in Egypt [114].

Evidence of traces of the uncommon *N*^6^-acyladenosine, phorioadenosine, **143**, has been reported by Farrugia et al. from a Southern Australian sponge *Phoriospongia* sp. [80].

## 5. Synthetic Approaches towards the Natural Alkylpurines and/or Bioactive Analogues

The secondary metabolites described here show interesting biological activities that sometimes are difficult to discover due to the small amounts that are obtained from their natural sources and the difficulties often found in collecting again the same organism. This fact limits their biological evaluation and the possibility for them to become drugs. Thus, the synthesis of either the natural products themselves or their analogues has become a challenge to the scientific community to determine the main structural features for the bioactivity and to analyse structure–activity relationships in these natural products.

In this sense, much synthetic work is devoted towards the synthesis of marine alkylpurines, particularly those with a diterpenoid moiety. This section is not intended to be exhaustive but rather to give an idea of the synthetic strategies used for this type of compounds. Furthermore, because most of this work have been well compiled in previous reviews [40,77,115], only some recently published approaches are considered here.

**Scheme 1:**
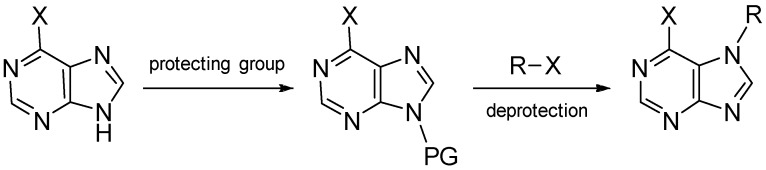
A general synthetic procedure towards 7-alkylpurines.

The main synthetic difficulties used to be associated to the regioselectivity during the alkylation of starting purines such as adenine, guanine or 6-chloropurine, bearing in mind that N9 is the most nucleophilic nitrogen and these natural products are usually substituted at N7. Thus, direct alkylation leads to both regioisomers predominating the N9-substituted isomer. To obtain the corresponding N7 isomers several strategies have been followed by different authors [40,77,115,116,117,118]. One of those relatively frequent strategies is to alkylate the N9 position with a protecting group, then to introduce the substituent at N7 and finally to deprotect N9, leading to the N7-substituted purines as summarised in the general Scheme 1. Other strategies include the Traube synthesis of purines [117] or the regioselective alkylation using alkylmagnesium reagents as recently described by Chen et al. [118].

**Scheme 2:**
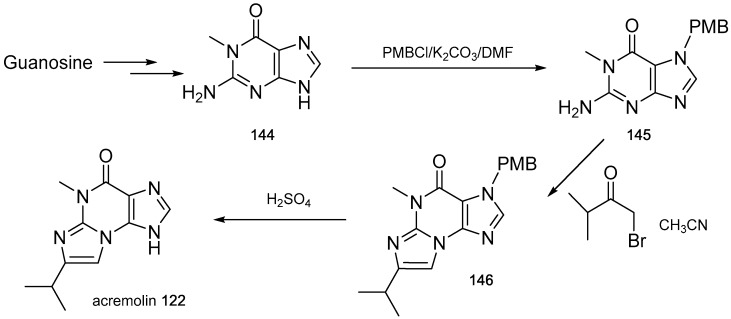
Molinski’s synthesis of acremolin, **122** [91].

Frequently most of these secondary metabolites are polymethylated/polyalkylated at more than one nitrogen atom and in those cases, dialkyl- and trialkylpurines can also be achieved selectively starting from purines already substituted at N3 or N9, which would direct the subsequent alkylation with the corresponding halide towards the other nitrogens. This strategy has been applied by Fujii et al., whose work on preparation of *N*-methylpurines stands out in the 1990s [119,120]. Sometimes, the initial substituents can be easily removed to obtain the desired purine analogue as it was applied to the synthesis of acremolin, **122** (Scheme 2) [91]. Molinski et al., starting from guanosine, obtained purine **144**, which was protected as its *para*-methoxybenzylderivative **145**; the separated N7 isomer was treated with the corresponding bromide to give the imidazole ring derivative **146** that, after deprotection with sulphuric acid or trifluoroacetic acid, gave the desired target acremolin, **122** [91].

**Scheme 3:**
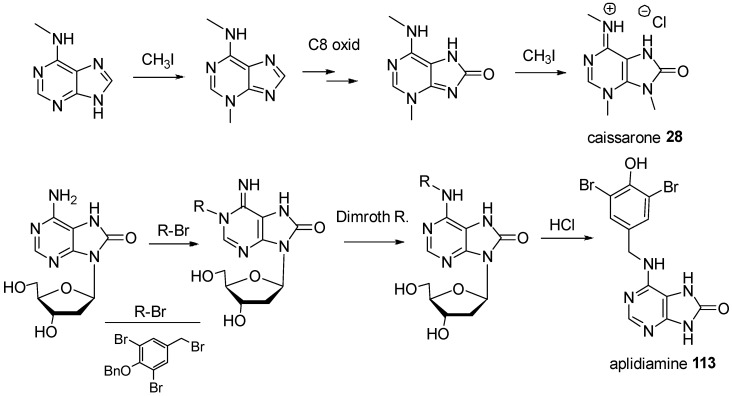
Fujii’s synthesis of caissarone, **28**, [120] and Itaya’s synthesis of aplidiamine, **113** [83].

The Dimroth rearrangement has been applied to the synthesis of *N*^6^-alkyladenines as several 8-oxoadenines [121,122,123], including simple purines as caissarone, **28** [34,120,123], and aplidiamine, **113** (Scheme 3) [83]. The introduction of the carbonyl group at C8 to give 8-oxo derivatives, has been achieved by different oxidation procedures, as very well summarised recently by Resendiz et al. [124] either on simple methylpurines or directly on nucleosides.

Perhaps the diterpenylpurines, such as agelasines, agelasimines and asmarines, are the most attractive synthetic targets due to the importance and variety of their biological activities. The first two have been synthesised by many authors, but there is not known yet a total synthesis of any natural asmarine.

**Scheme 4:**
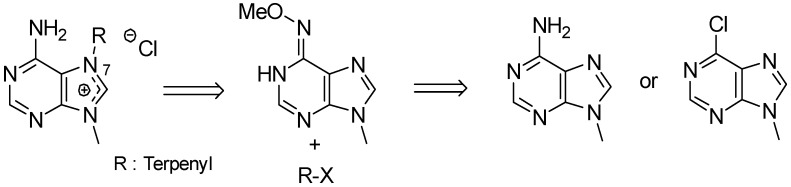
General retrosynthetic strategy for the synthesis of diterpenyladeninium derivatives.

One of the general synthetic strategies used for 9-methyl-7-diterpenyladeninium derivatives is the regioselective alkylation of *N*^6^-methoxy-9-methyladenine, obtained from 6-chloropurine or 9-methyladenine, with the corresponding terpenyl halide as represented in Scheme 4.

Gundersen et al. have done an extensive work on the synthesis of analogues of these marine secondary metabolites [40] (and references cited therein). They synthesised many analogues of agelasine E and F with simplified side chains [125] and diterpenyl halides obtained from other natural products such as geranylgeraniol or geranyllinalool [51]. The monocyclic side chain of agelasines E and F were obtained from geraniol and β-cyclocitral [126] and pulegone [127], respectively. They modified also the substitution on the purine C2 position [128,129,130]. All those analogues were evaluated for their antimicrobial, antiprotozoal, antifouling and biofilm inhibition and cytotoxicity against a panel of human cancer cell lines and it was found that the length of the terpenyl side chain is important for bioactivity and that the presence of a methyl in the purine 2-position increased the antimicrobial and antiprotozoal activity, while amino groups increase the cytotoxicity [40,130].

**Scheme 5:**
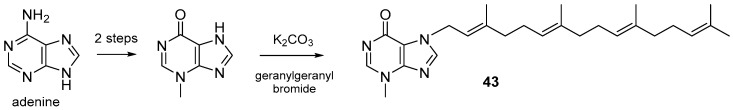
Gundersen’s synthesis of malonganenone J, **43** [131].

Continuing in the same line, Gundersen and co-workers have recently described the first synthesis of malonganenone J, **43**, starting from adenine to obtain 3-methylhypoxanthine that by alkylation with the corresponding geranylgeranyl bromide under mild conditions yielded malonganenone J, **43**, (Scheme 5) [131].

**Figure 32:**
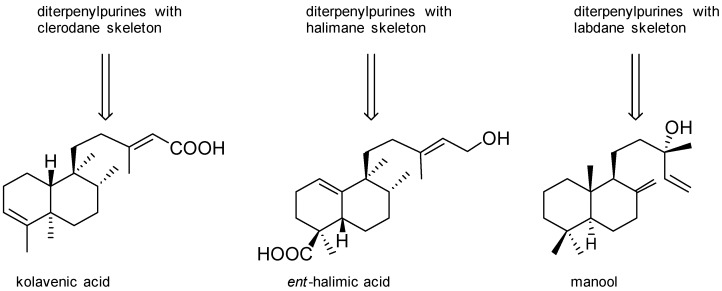
Structures of natural diterpenoids used in the synthesis of diterpenylpurines.

The synthesis of the agelasines and agelasimines with a bicyclic diterpenoid has been achieved by similar procedures and it was also compiled previously [40,115]. The bicyclic terpenoid side chain has been obtained from available natural diterpenoids or by total synthesis in a racemic [132,133] or chiral form [134,135]. Examples of natural diterpenoids used as starting material for the three types of diterpenoid skeletons are kolavenic acid [136], *ent*-halimic acid available in big quantities from its natural sources [66], or manool, which is commercially available [137] (Figure 32).

**Scheme 6:**
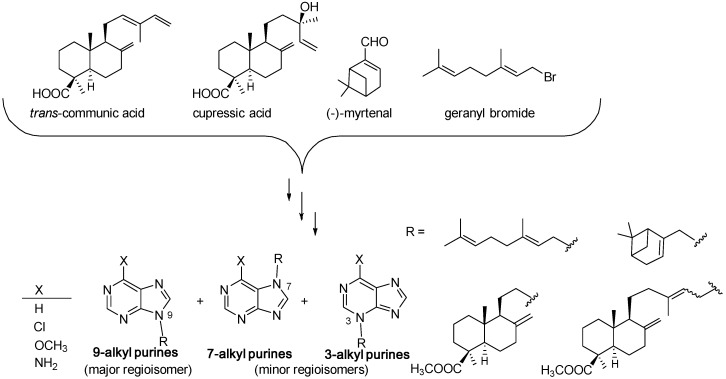
Terpenylpurines obtained from *trans*-communic and cupressic acids and monoterpenes.

A series of new neutral 9- and 7-terpenylpurines have been prepared by Castro et al. with monoterpenoid, sesquiterpenoid and diterpenoid side chains using commercial monoterpenoids such as myrtenal or geranylbromide and diterpenoids such as *trans*-communic acid (degradation of the side chain gave the sesquiterpenoid moiety) and cupressic acid isolated from their natural sources (Scheme 6) [138]. The cytotoxic evaluation against several human tumour cell lines indicated that the terpenyl chain induced cytotoxicity on simple purines, and this activity was higher as larger was the substituent, i.e., the best results were shown by the diterpenylpurines, in some cases with a very interesting low toxicity for non-tumour cells [138].

**Scheme 7:**
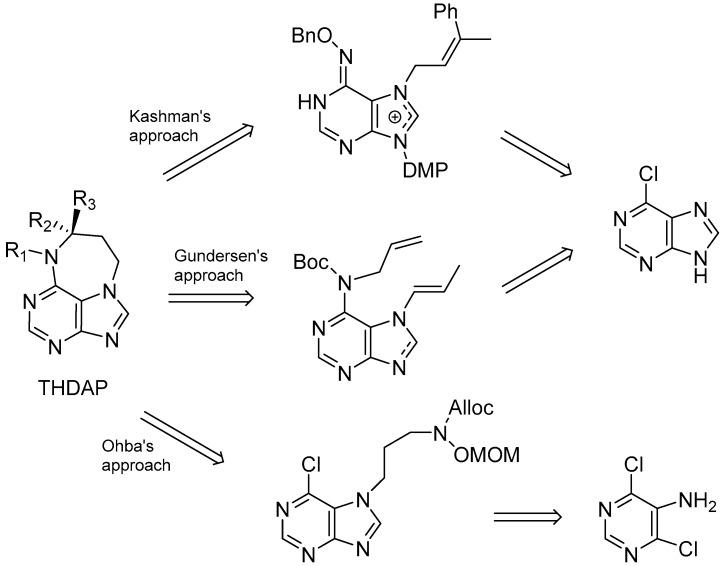
Retrosynthetic schemes for asmarines.

The other important group of diterpenylpurines are asmarines, for which no total synthesis has been reported yet; only several approaches towards the diazepinopurine system have been reported and they are summarised in Scheme 7 [40,115]. The tetrahydro[1,4]diazepino[1,2,3-*gh*]purine (THDAP) has been achieved mainly in three different ways. Kashman and co-workers used an aminomercuriation, iodocyclization and acid-catalyzed cyclization of an intermediate obtained from 6-chloropurine [139]. The same starting material was used by Vik and Gundersen to obtain an intermediate that by metathesis gave the required THDAP ring [140]. Finally, Ohba and Tashiro obtained the seven-membered ring by a palladium-promoted cyclization [141].

**Scheme 8:**
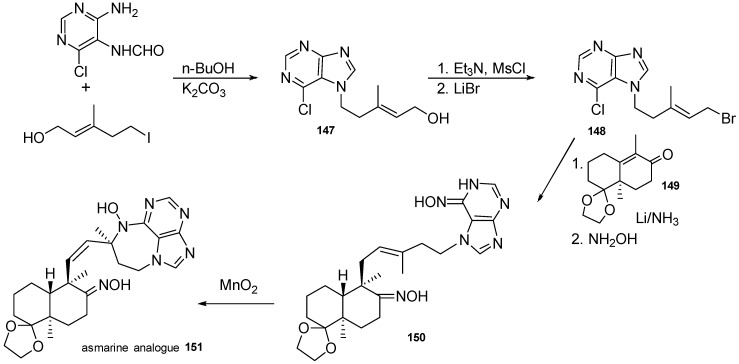
Synthesis of an advanced analogue of asmarine A by Shenvi [142].

Recently, Shenvi and co-workers [77,142] have obtained a very close analogue of asmarine A, **97**, by the cyclization through a nitrosopurine ene reaction as shown in Scheme 8. The starting formylpyrimidine was made to react with an alkyl iodide in basic conditions giving the alkylation and cyclization product, **147**, that was transformed into the corresponding bromide **148**. This bromide was made to react with the enolate of the methyl-Wieland-Miescher ketone, **149**, and then, by reaction with hydroxylamine, the corresponding dihydroxylimine **150** was obtained, as the ketone and the aryl chloride react as displayed in Scheme 8. Once the agelasine scaffold was obtained, oxidation of the *N*^6^-hydroxyaminopurine into a nitroso purine was achieved by treatment with MnO_2_ to give the desired tert-alkylmethylallylhydroxylamine **151**.

**Scheme 9:**
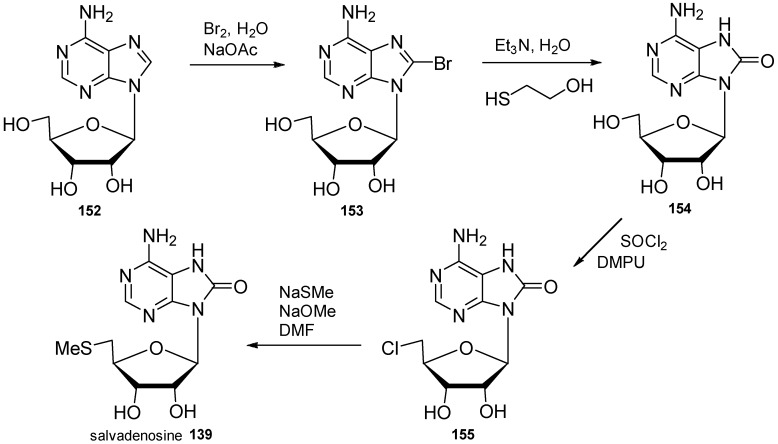
Molinski’s synthesis of salvadenosine, **139** [110].

Marine nucleosides have also been attractive targets for synthesis; their biological activities, synthesis and biosynthesis have been reported in a previous review [100] and only several recently reported synthesis have been considered here. That is the case of the synthesis of the 8-oxonucleoside salvadenosine, **139**, that was carried out by Molinski et al., starting with adenosine, **152**. Compound **152** was brominated at C8 to give **153**, transformed first into the corresponding 8-oxo-adenosine, **154**, and then into the chloroderivative **155** by treatment with thionyl chloride. Finally, displacement of the halogen with NaSMe, yielded salvadenosine, **139** (Scheme 9) [110].

**Scheme 10:**
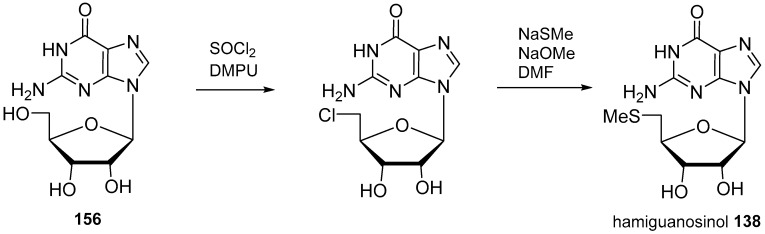
Molinski’s synthesis of hamiguanosinol, **138** [110].

The same displacement of the halogen with NaSMe was used by the same authors to synthesise hamiguanosinol, **138**, another marine nucleoside with a methylthio substituent in the sugar moiety from guanosine, **156**, and reassign its structure to the keto tautomer **138** (Scheme 10) [110].

**Scheme 11:**
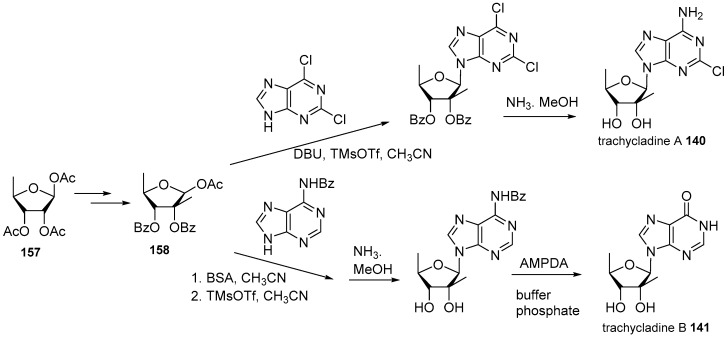
Synthesis of trachycladines A and B by Wu [143].

Another recent example is the total synthesis of the two marine nucleosides trachycladines A and B, **140**–**141**, performed by two different researchers in a very similar way as described below. The syntheses of Wu and co-workers (Scheme 11) started from a commercial triacetyl derivative of 5-deoxy-β-d-ribofuranose, **157**, that was transformed into the 2-methyl-dibenzoyl derivative **158**; ulterior coupling with 2,6-dichloropurine and treatment with ammonia in methanol gives trachycladine A, **140** [143]. For trachycladine B, **141**, *N*^6^-benzoyladenine was used as a base and the transformation into the hypoxanthine was achieved using an adenilate deaminase (AMPDA) as a biocatalyst.

**Scheme 12:**
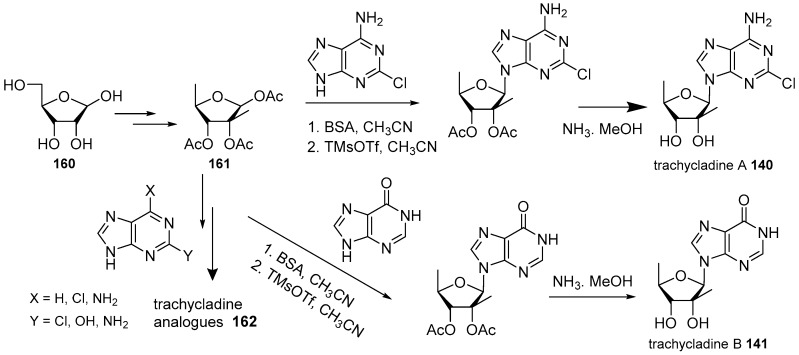
Synthesis of trachycladines A and B and analogues by Koumbis [144,145].

At the same time, Koumbis and co-workers [144] described the synthesis of both trachycladines A and B using similar methodology (Scheme 12), starting from d-ribose, **160**, through the 2-methyl triacetate **161** but using directly the corresponding unprotected nucleobase. This procedure has been improved by the same authors [145] and applied to the synthesis of several analogues (**162**) modified at the nucleobase in order to evaluate their cytotoxicity, which could not be evaluated previously for trachycladine B, **141**, because of the small amounts isolated from its natural source. The bioassays have shown the wide spectrum of antineoplastic activity for these compounds and the importance of the substituents in the purine ring, with trachycladine A being much more potent than trachycladine B.

## 6. Conclusions and Perspectives

Marine secondary metabolites containing a purine moiety alkylated at any nitrogen atom of the heterocycle are described in this review, considering the marine source and their biological properties. They have been isolated from a variety or marine organisms, mainly from sponges ascidians and gorgonians, but there are also a few examples isolated from other organisms such as a sea anemone, a nudibranch, two cone snail or one bryozoan, most of them collected at Caribbean, Okinawan, Australian and South China Sea. The purine system is derived from adenine, guanine, isoguanine and their 8-oxo-analogues and they have varied alkyl substituents ranging from methyl to diterpenoid chains. The largest group are the terpenylpurine derivatives that can bear acyclic, monocyclic or bicyclic diterpenoid side chains. The bicyclic ones are derived from clerodane, halimane or labdane skeletons. Diverse biological activities have been described for them such as anti-tumour, antibacterial, antifungal, anti-plasmodial, antifouling and biofilm inhibition, antiprotozoal, enzyme inhibition, or alteration of neuronal signalling among others, sometimes with very promising results. Although it is difficult to compare assays performed with different protocols, in general, those with a diterpenyl group are usually better cytotoxics than those with small chains while the methylpurines give better results on other bioassays. 

The marine organisms still are a good source of bioactive compounds derived from purine although the tiny amounts that are sometimes isolated limit their development as future drugs. Thus, more effort is necessary to develop good synthetic procedures that allow obtaining large quantities for biological assays including mechanisms of action. These efforts should not be directed solely towards the natural products themselves, but also to the preparation of simpler derivatives because, sometimes, they are even more potent than the original natural products.

## Figures and Tables

**Table 1:** Letter distribution of agelasines and related compounds according the diterpenoid skeleton.

Diterpenoid Skeleton	Agelasines
Clerodane type	agelasines A, B, G, H, I, K, L, M, N, P, Q, R, U, V
	ageloxime B
Halimane type	agelasines C, J, O, S
	agelasimines A, B
	gelasines A, B
Labdane type	agelasines D, T
	ageloxime D
Monocyclic diterpenoid	agelasines E, F
